# Proximal Fibular Osteotomy for Medial Joint Osteoarthritis of the Knee: A Prospective Cohort Study

**DOI:** 10.7759/cureus.19180

**Published:** 2021-11-01

**Authors:** Santosh Kumar, Shubham Srivastava, Sanjeev Kumar, Vikas Verma

**Affiliations:** 1 Department of Orthopaedic Surgery, King George's Medical University, Lucknow, IND; 2 Department of Paediatric Orthopaedic Surgery, King George's Medical University, Lucknow, IND

**Keywords:** akss score, vas, proximal fibular osteotomy, knee, osteoarthritis

## Abstract

Background and objective

Osteoarthritis (OA) is a polyarticular disease that most commonly afflicts the knee joint. Established operative treatment options for medial joint OA of the knee include high tibial osteotomy, unicompartmental knee arthroplasty, and total knee arthroplasty. Proximal fibular osteotomy (PFO) is a relatively new procedure for treating medial joint OA of the knee. The objective of this study was to describe the functional and radiological outcomes at one year in patients undergoing PFO for medial joint OA of the knee.

Materials and methods

The study included 21 patients with medial joint OA of the knee who underwent PFO. Visual analog scale (VAS) score, medial to lateral knee joint space ratio (ML ratio), Kellgren-Lawrence (KL) grade, and the American Knee Society Score (AKSS) (clinical and functional) were recorded preoperatively. VAS score, ML ratio, and AKSS (clinical and functional) were documented again at the three-month and one-year follow-ups.

Results

The mean age of the patients was 58.85 ±6.94 years; 12 (57.1%) were female and nine (42.9%) were males. The mean VAS score for pain decreased from 7.86 ±0.66 at baseline to 5.14 ±1.15 at three months (p<0.001) and 3.78 ±1.26 at one year (p<0.001). The mean clinical AKSS was 56.49 ±6.95 at baseline, which increased to 63.41 ±6.20 at three months (p<0.001) and 72.71 ±9.87 at one year (p<0.001). The mean functional AKSS at baseline was 48.24 ±14.31, which increased to 60.10 ±14.81 at three months (p<0.001) and 71.46 ±15.18 at one year (p<0.001). The mean ML ratio at baseline was 0.33 ±0.19, which increased to 0.41 ±0.20 at three months (p<0.01) and 0.51 ±0.22 at one year (p<0.001).

Conclusion

In patients who undergo PFO for OA of the knee, improvements in VAS score for pain, AKSS (functional and clinical), and ML ratio were observed to be maintained for a period of one year postoperatively.

## Introduction

Osteoarthritis (OA) is a polyarticular chronic degenerative disease of multifactorial etiology, and the most common joint to be afflicted is the knee [1]. A community-based study that involved people above 40 years of age from five different states of India reported the prevalence of the condition to be 28.7% [1]. OA of the knee joint is associated with advanced age, higher body mass index (BMI), female sex, and a sedentary lifestyle [1]. A study that included individuals above 60 years of age in the USA has estimated the prevalence of radiographic changes consistent with OA of the knee to be 37% [2]. The same study reported a prevalence of 12% for symptomatic OA of the knee. The lifetime risk of developing symptomatic knee OA has been reported to be 44.7% [3].

The severity of OA of the knee is classified as per the Kellgren-Lawrence (KL) grading, which categorizes it into five grades [4]. An important feature of OA of the knee is the compartment-specific narrowing of the joint space, which is associated with clinical manifestations of the disease [5]. Medial joint space narrowing is the most common type of narrowing [5]. Established operative treatment options for medial joint OA of the knee include high tibial osteotomy, unicompartmental knee arthroplasty, and total knee arthroplasty. Proximal fibular osteotomy (PFO) is a relatively new procedure for treating medial joint OA of the knee. Compared to other options, it is a simple, easy-to-do, and less invasive procedure, which requires only a small incision, limited dissection, and no internal fixation. Evidence regarding the functional and radiological outcomes of PFO for medial joint OA of the knee is scanty. In light of this, we planned this study to describe the functional and radiological outcomes of PFO in a group of patients over a one-year period.

## Materials and methods

This prospective cohort study was conducted among patients admitted to the Department of Orthopaedics, King George's Medical University (KGMU) in Lucknow, Uttar Pradesh, with medial joint OA of the knee. The study was conducted over a period of one year from August 2018 to July 2019. The diagnosis of OA of the knee was made using the clinical criteria laid down by the American College of Rheumatology (ACR) [6]. The ACR clinical criteria to diagnose OA of the knee are as follows: knee pain and at least three out of six of the following factors: age >50 years, morning stiffness <30 minutes, crepitus, bony tenderness, bony enlargement, and no palpable warmth. The study was approved by the Institutional Ethical Committee of KGMU vide reference no. 94 th ECM IIB- thesis/P-25. The procedures followed were in accordance with the ethical standards of the responsible committee on human experimentation (institutional and national) and with the Helsinki Declaration of 1975, as revised in 2000.

Preoperative and postoperative standard deviations (SD) of VAS scores for pain in patients undergoing PFO have been reported to be 1.5 and 2.34 respectively [7]. The required sample size was 18, which was calculated using the following formula:

N = (Z_α_ + Z_β_) (s_1_^2^ + s_2_^2^)/d^2 ^-^ ^where s_1 _= 1.5; s_2 _= 2.34; the SDs of the pre and postoperative VAS score in cases reported in the study by Wang et al., type I error at 5%; type II error at 10%; power at 90% [7].

However, we decided to enroll 21 patients considering any potential loss to follow up. We included patients with clinical OA of the knee diagnosed as per the ACR criteria. We excluded patients with incomplete records, patients with concomitant arthritis due to any other cause (rheumatoid, seronegative OA), patients with post-traumatic arthritis of the knee, patients with a history of ligament or meniscus injury of the knee, and patients with clinical valgus deformity of knees as measured using a goniometer.

**Figure 1:**
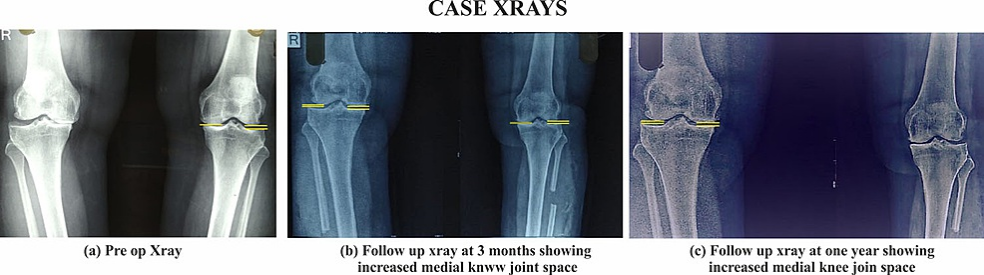
Images showing preoperative and postoperative X-rays

The medial joint space can be measured by measuring a vertical line drawn between a horizontal line passing through the lowest point of the femoral condyle and another horizontal line drawn through the tibial plateau on a full weight-bearing X-ray of the knee joint [7] (Figure 1). Lateral joint space can be determined by measuring a vertical line drawn between a horizontal line passing through the lowest point of the lateral femoral condyle and another horizontal line drawn through the tibial plateau on a full weight-bearing X-ray of the knee joint [7].

The American Knee Society Score (AKSS) scale developed by the American Knee Society is an examiner-dependent criterion [8]. It has two components: “clinical AKSS - knee score” and “functional AKSS - function score”. Clinical AKSS is derived through the physical examination of the knee. It evaluates pain (50 points), stability (25 points), and range of motion (25 points). Functional AKSS is derived by measuring the walking distance (50 points) and evaluating the act of climbing and descending stairs (50 points). Functional AKSS is affected by comorbidities and age [8,9]. On the other hand, clinical AKSS is not affected by comorbidities and age. It is also independent of functional AKSS [8].

Patients who were admitted to undergo PFO were offered enrolment provided they met the eligibility criteria of the study. Written informed consent was obtained from those who agreed to be enrolled. Patients enrolled in the study were assessed preoperatively for age, sex, duration of the disease, BMI, comorbidities, history of intra-articular injections, KL grade, VAS score for pain, AKSS (functional and clinical), and ML ratio.

**Figure 2:**
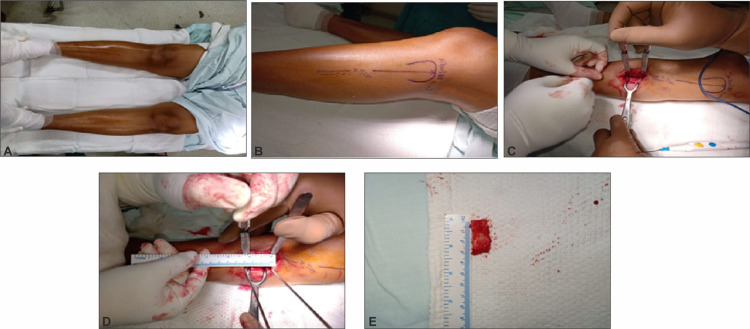
Images showing the operative technique

All patients were operated on exclusively by one author. Patients were placed in the supine position. Painting and draping were done from mid-thigh to the foot. Spinal anesthesia was used. A 5-6-cm long incision was made over the lateral aspect of the proximal fibula. The plane between the peroneal muscles and soleus was developed to expose the fibula. Hemostasis was achieved. Two osteotomies were done in the fibula using multiple drill holes in order to remove a 2-3-cm long segment of the fibula at a site 7-10 cm from the fibular head (Figure 2) [10]. Ankle and knee mobilization, as well as full weight-bearing, was allowed 24 hours after the surgery. Patients were discharged after the stitch removal. They were called for a follow-up at three months and at one year to record VAS scores for pain, AKSS (functional and clinical), and ML ratio based on full weight-bearing anteroposterior (AP) radiographs of the knee.

The data were entered into a Microsoft Excel spreadsheet. Analysis was done using SPSS Statistics version 16.0 (IBM, Armonk, NY). Categorical variables are presented in numbers and percentages (%) and continuous variables are presented as means ± SD and median. The normality of data was tested by the Kolmogorov-Smirnov test. If data were found to be non-normal, then a nonparametric test was used. The Wilcoxon signed-rank test was used for paired (pre and postoperative) data. Qualitative variables were compared using the Chi-square test/Fisher’s exact test as appropriate. A p-value of <0.05 was considered statistically significant.

## Results

Initially, 25 patients met the inclusion criteria. Two patients refused to be enrolled in the study, and 23 patients were eventually enrolled. However, two patients were lost to follow-up. Both the patients who were lost to follow-up had undergone bilateral PFO. Of the remaining 21, one patient had undergone unilateral PFO. Therefore, we are reporting the results of 21 patients and 41 knees. The mean age of the patients was 58.85 ±6.94 years (range: 48-74 years; median: 58 years). Twelve (57.1%) patients were female and nine (42.9%) were males. Nine patients (42.8%) had a history of intra-articular steroid injection; eight patients (38.09%) had a history of intra-articular hyaluronic acid injection, and two (9.5%) patients had a history of intra-articular platelet-rich plasma injection. Five patients had diabetes and 10 patients had hypertension. The distribution of patients as per KL grade is shown in Table 1.

**Table 1:** Distribution of knees as per KL grade KL: Kellgren-Lawrence

KL grade	Number of knees	%
1	2	4.85
2	22	53.65
3	14	34.14
4	3	7.31
Total knees	41	100.0

All postoperative wounds healed by primary intention. None of the patients had an infection. During the postoperative course, one patient reported paresthesia over the distribution of common peroneal nerve (CPN), and another patient developed sensory loss over the first webspace. Sensory loss, as well as paresthesia, resolved completely by three months. Two patients developed weakness of extensor hallucis longus, which also resolved completely by six months (Figure 2).

The mean VAS score for pain at baseline was 7.86 ±0.66, which decreased to 5.14 ±1.15 at three months (p<0.001; 34.60% decrease compared to baseline) and 3.78 ±1.26 at one year (p<0.0001; 51.50% decrease compared to baseline). The mean clinical AKSS at baseline was 56.49 ±6.95, which increased to 63.41 ±6.20 at three months (p<0.001; 12.25% increase compared to baseline) and 72.71 ±9.87 at one year (p<0.001; 28.71% increase compared to baseline). The mean baseline functional AKSS was 48.24 ±14.31, which increased to 60.10 ±14.81 at three months (p<0.001; 24.59% increase) and 71.46 ±15.18 at one year (p<0.001; 48.13% increase compared to baseline) (Tables 2, 3, 4).

The mean ML ratio at baseline was 0.33 ±0.19, which increased to 0.41 ±0.20 at three months (p<0.01; 24.24% increase over baseline) and 0.51 ±0.22 at one year (p<0.001; 54.52% increase over baseline) (Figure 1; Tables 2, 3, 4).

**Table 2:** Comparison of the means of clinical and radiological parameters at baseline and at three months (n=41 knees) VAS: visual analog scale; AKSS: American Knee Society Score; ML: medial to lateral knee joint space; SD: standard deviation

Variable	At baseline, mean ±SD	At three months, mean ±SD	P-value
VAS score	7.86 ±0.66	5.14 ±1.14	<0.001
Clinical AKSS	56.49 ±6.95	63.41 ±6,20	<0.001
Functional AKSS	48.24 ±14.31	60.10 ±14.81	<0.001
ML ratio	0.33 ±0.19	0.41 ±0.20	<0.01

**Table 3:** Comparison of the means of clinical and radiological parameters at three months and at one year (n=41 knees) VAS: visual analog scale; AKSS: American Knee Society Score; ML: medial to lateral knee joint space; SD: standard deviation

Variable	At three months, mean ±SD	At one year, mean ±SD	P-value
VAS score	5.14 ±1.14	3.78 ±1.26	<0.001
Clinical AKSS	63.41 ±6,20	72.71 ±9.87	<0.001
Functional AKSS	60.10 ±14.81	71.46 ±15.18	<0.001
ML ratio	0.41 ±0.20	0.51 ±0.22	<0.001

**Table 4:** Comparison of the means of clinical and radiological parameters at baseline and at one year (n=41 knees) VAS: visual analog scale; AKSS: American Knee Society Score; ML: medial to lateral knee joint space; SD: standard deviation

Variable	At baseline, mean ±SD	At one year, mean ±SD	P-value
VAS score	7.86 ±0.66	3.78 ±1.26	<0.001
Clinical AKSS	56.49 ±6.95	72.71 ±9.87	<0.001
Functional AKSS	48.24 ±14.31	71.46 ±15.18	<0.001
ML ratio	0.33 ±0.19	0.51 ±0.22	<0.001

## Discussion

In adults, OA of the knee joint is the most common cause of arthritis and is known to cause considerable pain and loss of function [7,8]. The choice of treatment depends on the age of the patient, stage of the disease, and the condition of the bones. Treatment options include arthroscopic debridement, high tibial osteotomy, PFO, unicompartmental knee arthroplasty, and total knee arthroplasty. High tibial osteotomy is indicated in young patients with medial joint OA and varus deformity of the knee [9]. In elderly patients with medial joint OA, the high tibial osteotomy is not the right choice due to concomitant osteoporosis causing a tibial plateau fracture [10]. Total knee arthroplasty provides effective pain relief and improved joint function. However, it is associated with several complications and may require revision [11]. Commonly used treatment options for medial joint OA in the young are high tibial osteotomy and unicompartmental knee arthroplasty. However, both are associated with several complications. High tibial osteotomy is associated with major complications, which include deep vein thrombosis, infection, incomplete correction, non-union, failure of internal fixation, peroneal nerve injury, and recurrence [12]. Unicompartmental knee arthroplasty is also associated with major complications such as injuries to the medial or lateral collateral ligaments of the knee, dislocation of the polyethylene bearing, degenerative arthritis in the other compartment, fracture of the medial proximal tibia, and dissociation of the prosthesis [13]. PFO is a relatively new procedure that has shown good results in medial joint OA of the knee.

The exact mechanism by which PFO provides pain relief and correction in varus alignment is still not clearly understood. With age, bone mass and bone density tend to decrease in load-bearing joints. The fibula provides support to the lateral condyle of the tibia, which leads to the non-uniform settlement of the tibial condyles with greater settlement and degeneration of the cartilage on the medial side [14]. Consequent to PFO, the support to the lateral half of the tibial plateau is reduced, which in turn may lead to the correction of varus deformity and lateral shift of the loading force [15]. The shift of the loading force to the less degenerated cartilage in the lateral half leads to pain relief and improvement in function [15]. Dong et al. put forward the theory of non-uniform settlement [16]. Prakash has proposed the theory of “too many cortices” as the mechanism behind improved function consequent to PFO [10]. Ground reaction vector readjustment has also been hypothesized to be the mechanism behind the success of PFO [17].

In our study, we observed significant pain relief in patients at three months, which continued to improve till one year. Significant pain relief following PFO as reported by us has been reported by several other studies [7,9,10]. Two studies have reported pain relief beyond one year [7,9]. In the study by Rai et al., the median follow-up period was 13.3 months, which was similar to the median follow-up of 13.38 months (range: 12-18 months) reported by Wang et al. [7,9]. The study by Wang et al. was a retrospective study and is therefore likely to have a recall bias [7].

Improvement in the knee and functional subsets of the AKSS as reported by us have been reported by other studies also [7,9]. Some studies have reported improvements in Oxford Knee Scores [10,8,18,19]. In our study, the clinical and functional AKSS improved from 56.49 ±6.95 to 72.71 ±9.87 and 48.24 ±14.31 to 71.46 ±15.18 respectively (p<0.001). The AKSS grades the outcome as poor (<60), fair (60-69), good (70-79), and excellent (>80) [18]. An excellent outcome in functional subsets of the AKSS (72.06 ±7.30 and 87.90 ±7.08) has been reported in another study [9]. However, that study involved patients who had comparatively higher preoperative clinical and functional scores (62.13 ±11.90 and 55.16 ±4.15) compared to our study (56.49 ±6.95 and 48.24 ±14.31) and that might be the reason for the higher postoperative scores. Wang et al. reported the AKSS outcome in the function subset in their study to be fair [7]. However, their study enrolled patients with comparatively lower AKSS. Preoperative knee society score has been reported to influence outcomes, which might explain the difference in outcomes between our findings and those of other authors [12].

A number of methods have been used to measure the radiological outcomes of PFO. These include the ML ratio [7,13,14], femorotibial angle (FTA) [7,15], condyle-plateau (CP) angle, settlement value, and absolute values of medial and lateral joint space [9,12,15]. An improved ML ratio as reported by us is consistent with other studies that have reported the outcomes at one year [7,13,14]. Studies using absolute values of medial and lateral joint space to measure the outcomes have reported significantly increased medial joint space and significantly decreased lateral joint space at one year [9,12] and at as long as 49.1 months [15]. A significant decrease in FTA has been reported at a mean follow-up of one year [9] and at as long as 49.1 months [15]. A significant decrease in CP angle has been reported at one-year follow-up in one study [12].

The present study is only the second one to have reported the outcome measures at three months and one year. This has helped us in ascertaining the temporal change in scores over a one-year period, i.e, improvements in VAS score, AKSS, and ML ratio appear as early as three months and continue to improve till one year. A recent study by Huda et al. published in 2021 has reported VAS score, Western Ontario and McMaster Universities Osteoarthritis Index (WOMAC) score, and FTA at three months and one year. They reported a significant reduction in VAS and WOMAC scores at three months. However, the reduction in VAS and WOMAC scores at six months and 12 months were not significant compared to the baseline. They did not report any significant difference in FTA at any point of the follow-up. The results of their study suggest that the benefits of PFO may not be long-lasting. This is in contrast with the improved radiological outcome reported in the present study and several other published studies [7,9,12-15]. A retrospective study by Yang et al. has shown that the improvement in radiological outcome continues beyond one year [15]. This raises the possibility of continued improvement even beyond one year. However, we are unable to comment on this possibility as this was beyond the scope of our study. Pain relief as well as improvement in functional knee score beyond one year has been reported in the literature [7,9]. A prospective study that follows up on patients for a longer duration will be able to throw light on the long-term effect of PFO.

Injury to the CPN has been reported to be a potential complication of PFO. CPN branches at a distance of 8.2 cm below the head of the fibula, and the maximal risk of injury to the nerve occurs within the proximal 15 cm of the fibula [20]. Yang et al. have reported CPN and superficial peroneal nerve injury in 1.8% cases each [15]. They also reported that 14.5% of their cases had a temporary weakness lasting up to four weeks. In the present study, the sensory loss, paresthesia, and weakness were also transient.

A limitation of our study is that we did not have a comparison group, which limited our ability to compare PFO with other available modalities of treatment for the medial joint OA of the knee. Another limitation is that we did not try to determine the factors that govern the outcomes in cases undergoing PFO. We recommend a prospective cohort study using regression modeling to determine the factors that govern the outcomes of PFO in cases of medial joint OA of the knee. A delineation of these factors will help the treating surgeon in choosing the right patients for PFO.

## Conclusions

PFO for medial joint OA of the knee is a short, safe, and simple procedure. However, the operating surgeon should be careful about the CPN perioperatively. PFO for the medial joint OA of the knee joint results in improved functional and clinical outcomes, decreased knee joint pain, and increased ML ratio. The changes are maintained for one year postoperatively. Opting for this procedure may help us potentially put off the more expensive and complicated total knee arthroplasty.
